# Editorial: High-Throughput Phenotyping in the Genomic Improvement of Livestock

**DOI:** 10.3389/fgene.2021.707343

**Published:** 2021-06-18

**Authors:** Fabyano Fonseca Silva, Gota Morota, Guilherme Jordão de Magalhães Rosa

**Affiliations:** ^1^Department of Animal Science, Federal University of Viçosa, Viçosa, Brazil; ^2^Department of Animal and Poultry Sciences, Virginia Polytechnic Institute and State University, Blacksburg, VA, United States; ^3^Center for Advanced Innovation in Agriculture, Virginia Polytechnic Institute and State University, Blacksburg, VA, United States; ^4^Department of Animal and Dairy Sciences, University of Wisconsin-Madison, Madison, WI, United States; ^5^Department of Biostatistics and Medical Informatics, University of Wisconsin-Madison, Madison, WI, United States

**Keywords:** automated phenotyping, computer vision, genome-enabled analysis, phenomics, precision livestock farming, animal breeding

Developments in the area of precision livestock farming (PLF) have promoted the use of high-throughput phenotyping or monitoring in animal breeding and genetics research and applications. For example, genome-enabled prediction, genome-wide association studies, and gene expression analysis have been revisited to accommodate a new opportunity introduced by high-throughput phenotyping (HTP) technologies. One of the most relevant challenges in this context is the handling of large-scale data provided by automated processes such as images collection, continuous real-time sensor-based measurements, and spectroscopy reports, among others. Genomic data analysis has been becoming more complex due to increasing phenotypic records from different data sources, as well as data structures and formats. The current Research Topic addresses this specific topic and includes studies on generating high-throughput phenotypes that were previously difficult or impossible to be measured manually, as well as on statistical or computational methodologies for integrating emerging types of phenotypes in breeding strategies. Such phenotypic traits can be used as indicator or novel traits for the genetic improvement of livestock populations for contemporary and pressing phenotypes or economic and societal importance such as animal health and welfare, environmental footprint, product quality, among others. The insights collected in this Research Topic offer new approaches for collecting, storing, and processing such HTP data.

Successful livestock breeding programs require large-scale and accurate phenotypic data, which are also critical for genomic dissection of complex traits. Digital image analysis, which represents the process of extracting meaningful information from images, can be used as input for imaging processing techniques with direct application for livestock phenotyping. Other technologies, such as activity monitor sensors, automatic feeding systems, and indirect biomarkers at the cellular and physiological levels can be used to provide a wide variety of novel phenotypes. Additionally, the infrared spectrometry has been gaining attention in PLF as a non-destructive measurement tool and a great resource for on-line analysis.

Automated image processing is critical in any application of computer vision to HTP as the size of generated image databases is often huge. Nye et al. proposed the Deep Automatic Phenotyping Segmentation (DeepAPS) framework to show how automatically parsed bull images from web-based sire catalogs together with pedigree data can be converted into useful information by inferring genetic parameters of several morphological measurements in dairy cattle. DeepAPS is based on deep learning, and represents a highly precise and automatic image processing algorithm to generate several phenotypic measures, including coat color and body conformational in dairy cattle.

Although image analysis and computer vision currently represent one of the most promising technologies to provide next-generation phenotypes for animal breeding purposes, there is a need to understand peculiarities behind them. Based on this issue, Fernandes et al. presented a detailed review focusing on a variety of computer vison technologies available as well as their applications to animal breeding programs. After a discussion on different strategies to process digital images, they review the main technologies available for imaging in animal and veterinary sciences, and present a number of applications of computer vision systems, including carcass and meat traits, behavior, health, among others.

According to Brito et al., genomic evaluation for animal welfare related traits using large-scale phenotyping is a suitable alternative for reliable selection under a commercial production system. The authors described the primary statistical and bioinformatic methods available for this aim, focusing on strategies for development of novel welfare indicator traits based on HTP, such as recording of movements based on wearable sensors and accelerometers. Sensor-based phenotypes such as movement capture based on IMU (Inertial Measurement Unit), sound analysis, and infrared thermography coupled with data science are paramount to translate animal welfare indicators into accurate genomic breeding values to be used in selective breeding to improve animal resilience at a commercial level. Bouwman et al. compared change point detection, local extreme approach, and gradient boosting machine for signal segmentation of turkey gait sequences collected from inertial measurement unit sensors. They reported gradient boosting machine was the most accurate method for signal segmentation to describe turkey gait. Temperament is a behavioral trait that is underutilized in animal breeding, mostly because the difficulties with systematic data collection. As reported by Yu et al., temperament could be considered as an indicator of production and efficiency in genetic selection. The authors developed a four-platform standing scale to objectively collect temperament data and proposed factor analytic modeling to investigate the underlying genetic interrelationships among temperament measures.

Infrared spectrometry has been also used to generate novel complex traits in livestock. Bresolin and Dórea presented a comprehensive review on mid- (MIR) and near-infrared (NIR) spectrometry for prediction of complex dairy and beef phenotypes, such as milk composition, feed efficiency, methane emission, fertility, energy balance, health status, and meat quality traits. The authors recommended the use of data mining tools to predict such phenotypes based on MIR and NIR. Additionally, Cecchinato et al. integrated experimental-lab measures, milk infrared spectra, and genomics to improve difficult-to-measure traits in dairy cattle populations. The authors concluded that Fourier-transformed infrared spectroscopy provided acceptable values of accuracy, making possible also the prediction for bulls without phenotyped progeny.

High-throughput phenotyping techniques offer a new opportunity to enhance genomic improvement of livestock, especially for novel phenotypes. The studies published in this Research Topic discuss excellent examples of their potential applications, and also point out for challenges and possible solutions. We encourage the scientific community and industry to read these interesting manuscripts and dive into this extremely exciting field of HTP and its applications in PLF and genetic improvement of livestock populations.

## Author Contributions

FFS, GM, and GJMR: conceptualization, writing, and review and editing. All authors contributed to the article and approved the submitted version.

## Conflict of Interest

The authors declare that the manuscript was written in the absence of any commercial or financial relationships that could be construed as a potential conflict of interest.

